# Follicular fluid meiosis activating sterol supplementation enhances oocyte maturation and fertilization in a microfluidic system: A lab trial study

**DOI:** 10.18502/ijrm.v22i10.17663

**Published:** 2024-12-02

**Authors:** Hossein Torkashvand, Ronak Shabani, Tayebe Artimani, Shamim Pilehvari, Mahdi Moghimi, Mehdi Mehdizadeh

**Affiliations:** ^1^Department of Anatomy, School of Medicine, Iran University of Medical Sciences, Tehran, Iran.; ^2^Reproductive Sciences and Technology Research Center, Department of Anatomy, School of Medicine, Iran University of Medical Sciences, Tehran, Iran.; ^3^Fertility and Infertility Research Center, Hamadan University of Medical Sciences, Hamadan, Iran.; ^4^School of Mechanical Engineering, Iran University of Science and Technology, Tehran, Iran.

**Keywords:** In vitro maturation, Microfluidic device, Follicular fluid, Sterol.

## Abstract

**Background:**

In vitro maturation (IVM) is a promising technique in assisted reproductive technologies, offering benefits such as reducing the risk of ovarian hyperstimulation syndrome.

**Objective:**

This study aimed to evaluate the effects of timed follicular fluid meiosis-activating sterol (FF-MAS) supplementation on the IVM of germinal vesicle oocytes using a dynamic microfluidic system.

**Materials and Methods:**

In this lab trial study, 266 germinal vesicle oocytes were collected from the Infertility Center of Fatemieh hospital, Hamedan, Iran between June 2023 and January 2024. The oocytes were allocated into 3 groups for dynamic microfluidic culture. Each group received culture medium at a flow rate of 0.36 µL/min for 24 hr through inlet A and FF-MAS supplementation through inlet B for 1, 2, and 6 hr. The study evaluated maturation and fertilization rates, embryo development, and mitochondrial status, which was assessed using the JC-1 mitochondrial membrane potential assay.

**Results:**

Maturation rates were significantly higher in the medium-term FF-MAS exposure (MTG) and long-term FF-MAS exposure groups compared to the short-term FF-MAS group (STG) (p < 0.05). Fertilization rates were also higher in the MTG and long-term FF-MAS group compared to the STG (p < 0.05). Embryo formation rates and the proportion of good-quality embryos were higher in the MTG compared to the STG (100% vs. 75%; p = 0.03) and (83.3% vs. 33.3%; p = 0.01), respectively. Mitochondrial peripheral distribution was significantly higher in the MTG than in the STG (p = 0.04).

**Conclusion:**

Optimizing FF-MAS exposure duration enhances IVM efficiency, offering a promising strategy to increase oocyte utilization in in vitro fertilization programs.

## 1. Introduction

According to recent statistics from the World Health Organization, approximately 17.5% of adults worldwide experience fertility problems (1). A significant portion of infertility cases, around 50%, are attributed solely to female-related factors (2). Rising rates attributed to adverse conditions like climate change, obesity and psychological stress have led to a rise in the use of assisted reproductive technologies (ART) among couples seeking assistance at reproductive clinics (3–6).

In vitro maturation (IVM) offers an attractive approach in human ART due to its significant advantages. It is a secure and cost-efficient method for generating embryos for in vitro fertilization (IVF) treatment (7). Unlike conventional methods, IVM does not rely on superovulation or synchronization, minimizing the risk of ovarian hyperstimulation syndrome (8). Despite its benefits, 15–30% of retrieved oocytes may remain immature at the germinal vesicle (GV) and GV breakdown (maturity metaphase I) stages following ovarian stimulation (9). This underscores the potential of IVM to improve oocyte utilization within IVF programs.

Enhancing the efficiency of the IVM process has become a key area of interest in reproductive research. Challenges such as low rates of oocyte maturation and embryo development necessitate careful selection of an appropriate culture system. This dual emphasis on innovation and meticulous system selection aims to optimize ART and improve outcomes for patients (10).

Researchers have been refining the structure and function of cells within static culture systems to more accurately replicate the dynamic environment of the female reproductive tract in mammals (11, 12). Following years of conducting IVM of immature GV oocytes in petri dishes, the microfluidic method was developed to create a system that closely resembles the in vivo environment (13). Microfluidics involves the study of systems that process and move tiny amounts of liquids using microchannels on a microscale (12). These approaches offer potential improvements in IVM success rates by precisely controlling the size and volume of microchannels.

In addition to refining physical conditions through microfluidics, various compounds have been noted to promote the maturation of oocytes (14, 15). Among these, follicular fluid meiosis-activating sterol (FF-MAS), stands out as a naturally occurring signaling molecule and a byproduct of the cholesterol synthesis pathway in oocytes (16). FF-MAS is found in high concentrations in the preovulatory follicular fluid of humans and has been demonstrated in vitro to induce meiosis in oocytes (17). In this study, we designed and fabricated a microfluidic device with dynamic capabilities to examine the effects of timed FF-MAS supplementation on the IVM of immature human GV oocytes, to improve maturation and fertilization rates. Our innovation lies in developing a dynamic microfluidic chip made from polydimethylsiloxane, which modulates fluid flow and biochemical gradients to mimic in vivo conditions essential for optimal oocyte maturation. This technology enabled precise control of FF-MAS exposure timing and concentration, creating a more physiologically relevant setting for oocyte maturation.

This dynamic microfluidic system represents a significant advancement in reproductive biology, offering precise control over environmental variables crucial for enhancing oocyte maturation rates and improving reproductive outcomes.

## 2. Materials and Methods 

### Participants selection 

In this lab trial study, conducted at the Infertility Center of Fatemieh hospital, Hamedan, Iran from June 2023 to January 2024, 298 immature GV oocytes were retrieved from 205 intracytoplasmic sperm injection (ICSI) cycles involving infertile women aged 20–35 yr without prior IVF failures.

Women aged ≥ 35 yr, poor responders, and those with a history of previous IVF failure were excluded from the study. Oocytes were selected based on normal appearance and donor age. Only GV oocytes with normal morphology were included; those with aberrations such as irregular shape, darkened cytoplasm diffuse granulation, abnormal perivitelline space, central cytoplasmic granulation, or different MI stages were excluded. Participants were stimulated using a standard gonadotropin-releasing hormone antagonist protocol (18).

### Oocyte collection 

Human chorionic gonadotropin (10,000 IU) (Choriomon, IBSA, Switzerland) was injected or administered 36 hr before oocyte retrieval. Oocytes were denuded by treating cumulus-oocyte complexes with 80 IU/mL hyaluronidase (Sigma, USA) and subsequently using glass pipettes to dissociate the cumulus cells mechanically. The denuded oocytes underwent evaluation for nuclear maturity under a stereo microscope (Olympus, Japan) (19). Following assessment, the oocytes were rinsed 2 times with G-Mops-V1 (Vitrolife, Sweden), and only those exhibiting normal GV status were selected for the subsequent.

### Preparation meiosis activating sterol (FF-MAS)

FF-MAS was purified from the human follicular fluid using Byskov's laboratory procedure (20). Briefly, 1 ml of follicular fluid was mixed with a 75% n-heptane and 25% isopropanol solution. Subsequently, 0.1 ml of 0.3 M NaH_2_PO_4_ (pH = 1.0) was added, which was vigorously shaken for 2 hr. The resulting solution was then centrifuged for 10 min at 2000 rpm to separate the organic phase containing sterols. This phase was collected, dried, and flushed with 2 ml of n-heptane, followed by centrifugation for 5 min at 2000 rpm. Supernatants obtained were dried and reconstituted in 150 mg of High-Performance Liquid Chromatographic (HPLC) eluent. These reconstituted samples were subsequently loaded onto a straight-phase HPLC column (ChromspherSi, 5 m, 250 mm × 4.6 mm) using a mixture of 99.5% n-heptane and 0.5% isopropanol (v:v), running at a flow rate of 1.00 ml/min. Specific sterols (lanosterol and T-MAS) were collected from the HPLC column. Reversed-phase separation was conducted by reconstituting the collected samples in acetonitrile and loading them onto a LiChrospher RP-8, 5 mm, 150 mm × 3.0 mm HPLC column. This column was run with a mobile phase comprising 93% acetonitrile and 7% water (v/v) at a flow rate of 1.00 ml/min, at room temperature. Quantification was done by comparing the eluted peaks from the samples with runs of standards (lanosterol, cholesterol, FF-MAS, and T-MAS) using known concentrations of these standards. FF-MAS samples were initially stored at -20 C in n-heptane under nitrogen. Before use, the n-heptane was evaporated with nitrogen gas, and the remaining MAS was dissolved in ethanol, and then added to the IVM culture medium.

### Microfluidic chip 

This microfluidic device is designed to replicate the natural growth environment of oocytes and embryos in vivo (21). Fabricated at the Mizan Microchip Technology Laboratory in Tehran, Iran, the device's 3D geometry was meticulously crafted using AutoCAD software and produced using a soft lithography technique. Constructed from polydimethylsiloxane, the polymer was chosen for its advantageous mechanical properties, elasticity, optical transparency, biocompatibility, and ease of manufacturing. After fabrication, the device was sterilized with ethylene oxide gas and stored in a clean plastic bag after each use.

**Figure 1:**
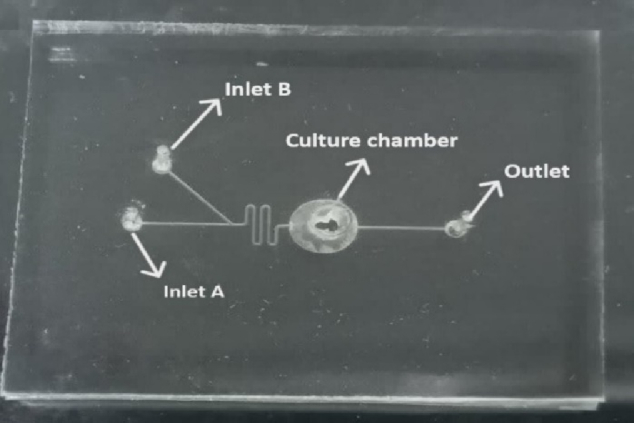
Different sections of the microfluidic device. x6 magnification.

The microfluidic device includes a channel that is 200 μ m across, 150 μ m in depth, and extends 8 mm overall. The compartment designed to collect mature oocytes measures 1300 µm in width. This device was equipped with 2 separate entry points: one for introducing the culture medium, and another for adding FF-MAS. There was also an exit point provided to facilitate the removal of both the culture medium and FF-MAS (Figure 1).

**Figure 2:**
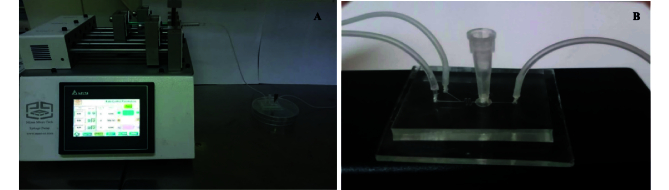
A, B) Image of the microfluidic device setup.

In this setup, GV oocytes were cultured within the chamber of a microfluidic device. A syringe pump (Mizan Micro Tech model: MMT-SP-102-Iran) was attached to Inlet A of the device using a silicone tube and connector, delivering the culture medium at a regulated flow rate of 0.36 µL/min. At the onset of the culture, the FF-MAS supplement was introduced to the oocytes through inlet B at the same flow rate of 0.36 µL/min by syringe pump for varying durations (Figure 2A, B). This flow rate was selected based on literature supporting its effectiveness in maintaining cell viability and minimizing shear stress, resulting in a flow velocity of 200 µm/s in the channel and approximately 4.52 µm/s within the chamber, ensuring gentle handling of the oocytes.

### Experimental design 

The allocation of GV oocytes into the experimental groups was achieved using random number tables, after which the oocytes were randomly divided into 3 groups. In all 3 groups, GV immature oocytes were cultured in fertilization media (Fert, ORIGIO, Denmark) under dynamic microfluidic conditions. For each round of cultivation, 2 oocytes were placed in the chamber of the microfluidic device, which served as the site for oocyte culture.

- **Short-term FF-MAS exposure group (STG)**

Culture medium: Infused through inlet A at a flow rate of 0.36 µL/min using a syringe pump (Mizan Micro Tech model: MMT-SP-102-Iran) for 24 hr.

FF-MAS supplement: Introduced through inlet B at a concentration of 10 µM and a flow rate of 0.36 µL/min for 1hr.

- **Medium-term FF-MAS exposure group (MTG)**

Culture medium: Infused through inlet A at the same flow rate of 0.36 µL/min using a syringe pump for 24 hr.

FF-MAS supplement: Introduced through inlet B at a concentration of 10 µM and a flow rate of 0.36 µL/min for 2 hr.

- **Long-term FF-MAS exposure group (LTG)**

Culture medium: Infused through inlet A at a flow rate of 0.36 µL/min using a syringe pump for 24 hr.

FF-MAS supplement: Introduced through inlet B at a concentration of 10 µM and a flow rate of 0.36 µL/min for 6 hr. All groups were maintained under the same experimental conditions: 37 C, 5% O ${\;}_{2}$ , and 6% CO ${\;}_{2}$ with high humidity.

### Evaluating the cellular toxicity of the microfluidic device

The assessment of cytotoxicity linked to the device was conducted through the human sperm viability assay. A total of 12 semen samples were obtained from individuals exhibiting normal sperm parameters. Sperm samples were prepared using a HEPES-buffered medium, and the resulting sperm suspensions were utilized for the human sperm viability assay. In the experimental group, the device was aseptically placed into a conical tube containing 1 ml of the sperm suspension. In contrast, the control group consisted of a conical tube with only 1 ml of spermatozoa, which was maintained at room temperature. Samples of the sperm suspension were taken at different time intervals: 0, 1, 2, 3, 4, 5, 24, 48, and 72 hr. With a sterile pipette, 10 μ l of the suspension was carefully transferred onto a glass slide. A coverslip was positioned on top, and the slide was subsequently observed under a microscope. The survival index (SI) was determined by assessing the percentage of progressively motile spermatozoa in the experiment. This was done by dividing the percentage of progressive motile spermatozoa in the test group by the percentage found in the control group at specified time intervals. Values of SI under 85% imply potential toxicity. This benchmark was applied in the present study to detect sperm toxicity (22).

### Fertilization and embryo development

The maturity of the oocytes was assessed after 24 hr by observing the presence of the first polar body under an inverted microscope (Nikon Co., Japan) (23). In the assessment of oocyte maturation, oocytes were classified as “degenerated” if they exhibited any of the following morphological abnormalities: darkened or fragmented cytoplasm, irregular shape, severe vacuolization, or the complete disintegration of the oocyte structure. “Arrested” oocytes were defined as those that failed to progress beyond the GV stage after 24 hr of in vitro culture. These oocytes exhibited a clear GV without evidence of further nuclear maturation, such as the absence of the first polar body (24). Oocytes matured in vitro underwent ICSI, and fertilization rates along with embryo development were evaluated and compared across 3 groups. Following ICSI, a fertilization status assessment was conducted 16–18 hr post-ICSI for each of the 3 groups. Subsequently, fertilized oocytes were cultured under conditions similar to those of the initial oocyte culture. However, the SAGE 1-step media (ORIGIO, Denmark) used for the embryos did not contain the FF-MAS supplement. Embryo quality was assessed approximately 44–48 hr after ICSI using Hill's ranking system. In this system, embryos are classified into 4 grades based on blastomere morphology and the extent of fragmentation: grade A was considered high-quality, grade B good-quality, grade C intermediate, and grade D poor. Embryos with A or B grades are known as “good quality embryos", those with C are considered “intermediate”, and those with D are classified as “poor” quality embryos (25).

### Rate of mitochondrial activity and mitochondrial distribution

The mitochondrial status of oocytes was assessed using the JC-1 assay kit for mitochondrial membrane potential (Cayman Chemical Co., USA). For the preparation of the JC-1 staining solution, the stock solution was diluted in Hams F10 medium at a ratio of 1:10. Subsequently, a working solution was made by combining 5 μ L of the staining solution with 150 μ L of Hams F10 medium. Oocytes were placed into droplets of the JC-1 working solution using an ICSI pipette and incubated in the dark for 30 min at 37 C. The oocytes were then observed with a fluorescence microscope (Olympus, Japan) (26). Inactive oocytes appeared green (low mitochondrial membrane potential), while active oocytes appeared red (high mitochondrial membrane potential). Mitochondrial activity rates and distribution patterns were compared among the 3 experimental groups.

### Ethical Considerations

This study was approved by the Ethics Committee of the Iran University of Medical Sciences, Tehran, Iran (Code: IR.IUMS.FMD.REC.1401.314). Participants provided informed written consent after receiving detailed information about the study's objectives, procedures, and potential benefits through verbal and written explanations. The voluntary nature of participation was emphasized; ensuring participants understood their right to withdraw at any time without consequences.

### Statistical Analysis

The findings are expressed as mean ± standard deviation. Fisher's exact test was employed where appropriate. The data distribution was assessed using the D'Agostino-Pearson test. All hypotheses were two-tailed, with statistical significance set at p < 0.05. The statistical analysis was performed utilizing GraphPad Prism version 8.4.2.

## 3. Results 

### Cellular toxicity of the microfluidic device

Table I displays the outcomes of the human sperm viability assay performed in this study. The findings indicated that the SI exceeded 85% across various assessment periods, confirming that the microfluidic device is safe for use and does not exhibit any toxic effects.

Table I displays the outcomes of the human sperm viability assay performed in this study. The findings indicated that the SI exceeded 85% across various assessment periods, confirming that the microfluidic device is safe for use and does not exhibit any toxic effects.

### Assessment of oocyte maturation, fertilization, and embryo development

A total of 298 immature oocytes at the GV stage were retrieved from a standard 205 ICSI stimulated cycles. The morphological assessment was conducted through microscope visualization, resulting in the exclusion of 32 GV oocytes (10.7%) due to abnormalities such as irregular shape, dark cytoplasm, diffuse granulation, perivitelline space abnormalities, central cytoplasmic granulation, and cumulus-oocyte complex irregularities. Consequently, 266 oocytes (89.3%) met the inclusion criteria based on normal morphology. Analysis of the culture conditions for the 266 GV oocytes across 3 groups revealed maturation rates of 54.6%, 69.4%, and 71.4% for STG, MTG, and LTG, respectively. Statistical analysis indicated a significant difference in maturation rates between MTG and STG (69.4% vs. 54.6%; p = 0.04) and between LTG and STG (71.4% vs. 54.6%; p = 0.02). Moreover, significant differences in fertilization rates were observed in MTG compared to STG (70.5% vs. 42.8%; p = 0.03) and in LTG compared to STG (68.4% vs. 42.8%; p = 0.04). Additionally, a statistically significant difference was observed in embryo formation rates between MTG and STG (100% vs. 75%; p = 0.03). A statistically significant increase was observed in the percentage of good-quality embryos in MTG compared to STG (83% vs. 33.3%; p = 0.01) (Table II).

### Assessment of rate of mitochondrial activity and active mitochondrial distribution patterns

**Figure 3:**
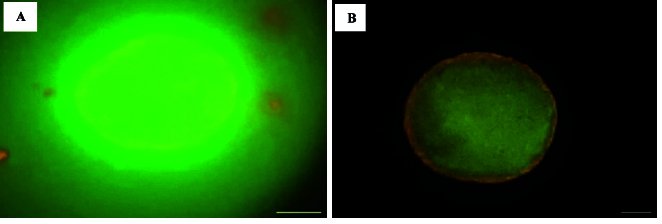
Fluorescence microscopy images (x400 magnification) using JC-1 dye showing the rate of mitochondrial activity and the distribution patterns of mitochondria during the maturation of oocytes. A) The inactive oocytes appeared green, indicating low mitochondrial membrane potential. B) The active oocytes appeared red, indicating high mitochondrial membrane potential, active mitochondria show a peripheral distribution pattern in MII oocytes. Scale bar represents 50 µm.

The impact of culture conditions on the rate of mitochondrial activity and active mitochondrial distribution patterns were assessed using JC-1 dye. The results indicated that the rate of mitochondrial activity in the matured oocytes did not show any significant difference between the different groups (Table III, Figure 3A, B).

Specifically, the percentage of active mitochondria was 80% in STG, 92% in MTG, and 90.9% in LTG, indicating that while MTG had the highest activity rate, it did not show a statistically significant difference with the STG (p = 0.4) and LTG (p = 0.9). 3 distinct patterns of active mitochondria were observed: peripheral, semiperipheral, and diffused. The peripheral arrangement showed active mitochondria located primarily in the cortical area of the oocytes. The diffused pattern demonstrated that active mitochondria were evenly distributed throughout the cytoplasm. Meanwhile, the semiperipheral arrangement, depicted an intermediate state, with active mitochondria situated between the cortical and central areas (27). The results demonstrated that after IVM, active mitochondria exhibited a higher percentage of the peripheral distribution pattern across all 3 groups. Notably, the peripheral mitochondrial distribution in the MTG showed a significant increase compared to the STG (Table III, Figure 3B).

**Table 1:** Assay of human sperm survival for testing the toxicity of a microfluidic device

[1.2in,lr]**Parameters** **Time**	**0 hr**	**1 hr**	**2 hr**	**3 hr**	**4 hr**	**5 hr**	**24 hr**	**48 hr**	**72 hr**
**Control**	86%	83%	80%	76%	74%	70%	65%	56%	50%
**Experimental **	88%	83%	78%	77%	72%	65%	61%	55%	48%
**Survival index**	1.02	1.00	0.97	1.01	0.97	0.92	0.93	0.98	0.96
**Success**	✓	✓	✓	✓	✓	✓	✓	✓	✓
**Failure**	-	-	-	-	-	-	-	-	-

**Table 2:** Results of IVM, fertilization, and embryo formation rates in human oocytes

**Variables**		**STG**	**MTG**	**LTG**	**P-value**
**No. of oocytes**		97	85	84	-
**Matured **		53 (54.6)	59 (69.4)	60 (71.4)	0.04^a^ 0.02^b^ 0.86 ${\;}^{\text{c}}$
**Arrested **		33 (34)	22 (25.8)	19 (22.6)	0.25^a^ 0.10^b^ 0.72 ${\;}^{\text{c}}$
**Degenerated **		11 (11.3)	4 (4.7)	5 (5.9)	0.11^a^ 0.29^b^ 0.74 ${\;}^{\text{c}}$
**Fertilization rate**		12 (42.8)	24 (70.5)	26 (68.4)	0.03^a^ 0.04^b^ 0.9 ${\;}^{\text{c}}$
**Embryo formation (2 cell) rate**		9 (75.0)	24 (100)	25 (96.1)	0.03^a^ 0.08^b^ 0.9 ${\;}^{\text{c}}$
**Embryo quality**					
	**Good quality**	3 (33.3)	20 (83.3)	17 (68.0)	0.01^a^ 0.11^b^ 0.32 ${\;}^{\text{c}}$
	**Intermediate**	4 (44.4)	3 (12.5)	7 (28.0)	0.06^a^ 0.42^b^ 0.28 ${\;}^{\text{c}}$
	**Poor quality**	2 (22.2)	1 (4.16)	1 (4.0)	0.17^a^ 0.16^b^ 0.9 ${\;}^{\text{c}}$
Data presented as n (%). Fisher's exact test. IVM: In vitro maturation, STG: Short-term FF-MAS exposure group, MTG: Medium-term FF-MAS exposure group, LTG: Long-term FF-MAS exposure group. a: STG vs. MTG, b: STG vs. LTG, c: MTG vs. LTG					

**Table 3:** Rate of mitochondrial activity and distribution patterns of mitochondria in MII and active oocytes

**Variables**	**STG**	**MTG**	**LTG**	**P-value**
**No. of oocytes MII**	25	25	22	-
**Active oocytes**	20 (80.0)	23 (92.0)	20 (90.9)	0.41^a^ 0.42^b^ 0.9 ${\;}^{\text{c}}$
**Peripheral**	10 (50.0)	21 (91.3)	18 (90.0)	0.04^a^ 0.05^b^ 0.9 ${\;}^{\text{c}}$
**Semiperipheral**	6 (30.0)	2 (8.69)	1 (5.0)	0.11^a^ 0.09^b^ 0.9 ${\;}^{\text{c}}$
**Diffused**	4 (20.0)	0 (0.0)	1 (5.0)	0.06^a^ 0.34^b^ 0.46 ${\;}^{\text{c}}$
Data presented as n (%). Fisher's exact test. MII: Metaphase II, STG: Short-term FF-MAS exposure group, MTG: Medium-term FF-MAS exposure group, LTG: Long-term FF-MAS exposure group. a: STG vs. MTG, b: STG vs. LTG, c: MTG vs. LTG				

## 4. Discussion

In this study, we aimed to evaluate the impact of timed FF-MAS supplementation on the maturation and fertilization rates of immature human GV oocytes using a dynamic microfluidic system. We specifically investigated the effects of different exposure durations to FF-MAS on oocyte maturation and subsequent fertilization outcomes. Our findings demonstrated that timed FF-MAS supplementation significantly improved the maturation and fertilization rates of immature human GV oocytes in vitro. A 2-hr exposure (MTG) led to the most substantial improvements in maturation and fertilization rates, aligning with the natural in vivo exposure time. MTG and LTG, which were exposed to FF-MAS for 2 and 6 hr, respectively, exhibited higher oocyte maturation and fertilization rates compared to the STG, which was exposed for 1 hr. It is well-established that FF-MAS plays a crucial role in the resumption of meiosis and oocyte maturation by modulating the microenvironment and providing essential developmental signals. These improvements align with previous studies that also demonstrated the positive effects of FF-MAS on oocyte maturation and quality (20, 28). Despite the inherent challenges associated with immature oocytes-such as reduced quality due to smaller follicles, lack of meiotic competence, or insufficient hormonal stimulus-our study demonstrates that FF-MAS supplementation can enhance oocyte maturation even in the absence of cumulus cells. This highlights FF-MAS's potential to significantly improve oocyte quality, providing a valuable advancement in IVM techniques.

Moreover, the fertilization rate significantly increased in MTG and LTG compared to STG. This improvement can be attributed to the enhanced nuclear maturation facilitated by FF-MAS, which mimics in vivo conditions and supports the completion of meiotic maturation in vitro. This maturation is crucial for successful fertilization post-ICSI. Our results suggest that the duration of FF-MAS supplementation, when closely mimicking in vivo conditions, is positively associated with the formation of high-quality embryos based on Hill's classification. The increased concentration of FF-MAS in the ovaries during nuclear and cytoplasmic maturation suggests its physiological role in these processes. These findings are consistent with earlier research that has highlighted the beneficial effects of FF-MAS on oocyte quality and subsequent embryonic development (29).

Mitochondria, the most abundant organelles in the cytoplasm, provide ATP for oocytes and play crucial roles in motility, maintenance of cellular homeostasis, and regulation of cell survival (30). In this study, we evaluated mitochondrial distribution patterns and membrane potentials using the fluorescent marker JC-1, following previously established protocols (31). Our results indicated that FF-MAS supplementation did not significantly alter mitochondrial activity in metaphase II oocytes from different culture groups. However, we observed that a higher percentage of MII oocytes in MTG exhibited peripheral mitochondrial distribution compared to the STG, a feature often associated with better developmental potential. Mitochondrial distribution is a crucial indicator of oocyte quality, as mitochondria supply the ATP needed for cellularprocesses and are involved in signaling pathways that regulate oocyte maturation and early embryonic development. These findings are consistent with previous studies, which demonstrates that mitochondrial redistribution is closely linked with oocyte maturation. The lack of such redistribution is often associated with incomplete cytoplasmic maturation and reduced developmental competence (32, 33). Although these observations are preliminary, they highlight the need for further research to clarify the role of FF-MAS in modulating mitochondrial function during oocyte maturation. Future studies could investigate the molecular mechanisms underlying these changes, and explore whether enhancing mitochondrial redistribution could further improve oocyte developmental potential.

The improvements observed in oocyte maturation and fertilization rates are primarily attributable to the timed supplementation of FF-MAS. While the microfluidic system enhanced the experimental conditions by providing precise control over variables such as FF-MAS concentration and environmental parameters, its role was largely supportive. Previous studies have established that FF-MAS alone can effectively improve oocyte maturation, indicating that the major contributing factor to our results is FF-MAS itself (20, 28). The microfluidic system facilitated a more controlled environment, thereby optimizing the effects of FF-MAS but not serving as the primary driver of the observed improvements. Moreover, the microfluidic system's ability to simulate in vivo-like conditions allowed for more natural and continuous interaction between FF-MAS and the oocytes, potentially amplifying the biological effects of FF-MAS (13). This setup minimized external fluctuations and ensured that the observed improvements were directly attributable to the timed supplementation of FF-MAS, reinforcing its role as the key factor in enhancing oocyte maturation and fertilization rates.

### Strengths and limitations

The study's findings regarding the potential of IVM to enhance the quantity and quality of immature oocytes in infertile women carry significant consequences for future research directions and clinical applications. Nonetheless, certain limitations should be acknowledged. The relatively small sample size suggests that additional research with larger participant groups is essential to validate these findings. Additionally, the mechanisms by which FF-MAS impacts nuclear and cytoplasmic maturation remain unclear, necessitating more in-depth exploration of the molecular pathways involved.

Despite these limitations, the study provides valuable insights. Investigating the molecular pathways influenced by FF-MAS could lead to a deeper understanding and more targeted interventions in oocyte maturation protocols. Clinically, the study supports the idea that IVM with an in vivo-like approach may serve as a viable and effective treatment for women with multiple immature oocytes. This indicates that IVM has the potential to serve as a notable substitute for standard practices like IVF and other assisted reproductive methods.

## 5. Conclusion

Our study provides new insights into the impact of FF-MAS supplementation on oocyte maturation and fertilization rates using a dynamic microfluidic system. We found that simulating in vivo conditions with FF-MAS supplementation significantly enhances oocyte maturation and fertilization rates. Specifically, replicating the brief, physiological exposure of FF-MAS led to optimal improvements in cytoplasmic maturation, including better peripheral distribution of mitochondria, and subsequently improved embryo quality. These findings underscore the importance of mimicking natural conditions to achieve the best outcomes in vitro and highlight FF-MAS potential to enhance oocyte quality and developmental success in ART.

##  Data Availability

Data supporting the findings of this study are available upon reasonable request from the corresponding author.

##  Author Contributions

H. Torkashvand, R. Shabani, T. Artimani, Sh. Pilehvari, and M. Mehdizadeh had full access to all of the data in the study and takes responsibility for the integrity of the data and the accuracy of the data analysis. Concept and design: H. Torkashvand, M. Moghimi, M. Mehdizadeh. Acquisition, analysis, or interpretation of data: H. Torkashvand, R. Shabani, T. Artimani and Sh. Pilehvari. Drafting of the manuscript: All authors. Critical revision of the manuscript for important intellectual content: All authors. Statistical analysis: H. Torkashvand, T. Artimani, Sh. Pilehvari. Supervision: H. Torkashvand, M. Mehdizadeh.

##  Conflict of Interest

The authors declare that there is no conflict of interest.
